# Structural and Functional Analyses of DNA-Sensing and Immune Activation by Human cGAS

**DOI:** 10.1371/journal.pone.0076983

**Published:** 2013-10-07

**Authors:** Kazuki Kato, Ryohei Ishii, Eiji Goto, Ryuichiro Ishitani, Fuminori Tokunaga, Osamu Nureki

**Affiliations:** 1 Department of Biophysics and Biochemistry, Graduate School of Science, The University of Tokyo, Bunkyo-ku, Tokyo, Japan; 2 Global Research Cluster, RIKEN, Wako, Saitama, Japan; 3 Laboratory of Molecular Cell Biology, Institute for Molecular and Cellular Regulation, Gunma University, Maebashi, Gunma, Japan; Juntendo University School of Medicine, Japan

## Abstract

The detection of cytosolic DNA, derived from pathogens or host cells, by cytosolic receptors is essential for appropriate host immune responses. Cyclic GMP-AMP synthase (cGAS) is a newly identified cytosolic DNA receptor that produces cyclic GMP-AMP, which activates stimulator of interferon genes (STING), resulting in TBK1-IRF3 pathway activation followed by the production of type I interferons. Here we report the crystal structure of human cGAS. The structure revealed that a cluster of lysine and arginine residues forms the positively charged DNA binding surface of human cGAS, which is important for the STING-dependent immune activation. A structural comparison with other previously determined cGASs and our functional analyses suggested that a conserved zinc finger motif and a leucine residue on the DNA binding surface are crucial for the DNA-specific immune response of human cGAS, consistent with previous work. These structural features properly orient the DNA binding to cGAS, which is critical for DNA-induced cGAS activation and STING-dependent immune activation. Furthermore, we showed that the cGAS-induced activation of STING also involves the activation of the NF-κB and IRF3 pathways. Our results indicated that cGAS is a DNA sensor that efficiently activates the host immune system by inducing two distinct pathways.

## Introduction

In the innate immune system, germline-encoded pattern recognition receptors (PRRs) recognize both non-self products, pathogen-associated molecular patterns (PAMPs), and self products, damage-associated molecular patterns (DAMPs), to activate signaling pathways resulting in the production of proinflammatory cytokines or type I interferons (IFNs) [1]. Cytosolic DNAs, derived from invading pathogens and host genomes, are strong activators of the innate immune system as PAMPs and DAMPs, respectively [2]. So far, several types of PRRs have been reported as cytosolic DNA sensors [3-12]. Recent studies have shown that stimulator of interferon genes, STING (also named MPYS, MITA, or ERIS), plays a central role in the signaling cascades of cytosolic DNA sensing [13,14]. The activation of STING by cytosolic DNA triggers the TBK1-IRF3 pathway, to induce the expression of IFNs, as well as the NF-κB transcription pathway, to produce cytokines [15]. Although pathogenic cytosolic DNA activates STING-dependent pathways, STING itself binds to DNA with very low affinity *in vitro*, suggesting the requirement of another PRR for DNA sensing [16]. Recent studies identified cyclic GMP-AMP synthase (cGAS) as a cytosolic DNA PRR, and its product, cyclic GMP-AMP (cGAMP), binds to STING as a second messenger, resulting in the activation of the STING-dependent IRF3 pathway [17,18].

Quite recently, three groups have solved the crystal structures of mouse cGAS alone and in complex with DNA/nucleotides, porcine cGAS alone and in complex with DNA/nucleotides, and human cGAS alone, respectively [19-21]. Together, these results indicated that upon DNA binding, a conformational change occurs that is essential for cGAMP production.

Here, we report the structure of human cGAS in a novel crystal form. On the basis of structural comparisons with the mouse and porcine cGAS structures and functional analyses, we discuss the importance of the common structural features, along with the relevance of DNA detection, for the STING-dependent immune activation.

## Materials and Methods

### Protein preparation and crystallization

The gene encoding human cGAS (residues 161-522) was inserted into a modified pE-SUMO Vector (LifeSensors), with insertion of the sequence cleaved by TEV protease between the N-terminal SUMO tag and the multicloning site. The protein was overexpressed in *Escherichia coli* Rosetta2 (DE3) (Novagen), and purified by chromatography on Ni-NTA (Qiagen), HiTrap Heparin and Superdex 200 gel filtration columns (GE Healthcare). The cGAS mutants were prepared by a PCR-based method, and the sequences were verified by DNA sequencing. The mutants were purified according to a protocol similar to that used for wild type cGAS. Crystallization was performed at 20°C by the sitting drop vapor diffusion method. Crystals were obtained in a buffer consisting of 18% PEG3350, 0.2 M ammonium nitrate, 0.5 M NaCl, and 0.02 mM CYMAL-7.

### Data collection, structure determination, and refinement

Crystals were cryoprotected in the crystallization buffer supplemented with 30% glycerol. X-ray diffraction data were collected on beamline BL41XU at SPring-8 (Hyogo, Japan), using an MX225HE detector. Data were processed and scaled with the HKL2000 program package (HKL Research Inc.). The structure was solved by the molecular replacement method with the MOLREP program [22], using the complex structure of mouse cGAS with DNA (PDB ID 4K96) [19] as the search model. Model building and refinement were performed using COOT [23] and PHENIX [24], respectively. Data collection and refinement statistics are summarized in Table 1. Coordinates and structure factors have been deposited in the Protein Data Bank (PDB) under accession code 4MKP.

**Table 1:** Data collection and refinement statistics.

	Human cGAS
**Data collection**	
Space group	*P*2_1_2_1_2
Cell dimensions	
*a*, *b*, *c* (Å)	123.5, 48.31, 59.57
α, β, γ (°)	90, 90, 90
Resolution (Å)	50.00-1.95 (1.98-1.95)
*R*_sym_	0.086 (0.790)
*I* / σ*I*	34.2 (2.2)
Completeness (%)	97.7 (94.8)
Redundancy	6.8 (6.6)
**Refinement**	
Resolution (Å)	42.9-1.95
Number of reflections	25,996
*R*_work_/*R*_free_	0.206/0.252
Number of atoms	2,671
Protein	2,556
Ligand/ion	1
Water	114
*B*-factors	
Protein	51.3
Ligand/ion	26.0
Water	46.1
R.m.s. deviations	
Bond lengths (Å)	0.010
Bond angles (°)	1.197
Ramachandran plot (%)	
Favored	96.5
Allowed	3.16
Outlier	0.32

Data for the highest-resolution shell are shown in parentheses.

### DNA-binding experiment

The cGAS wild type and mutant proteins were mixed with biotinylated ISD (Interferon stimulatory DNA, Sense strand sequence 5′-TACAGATCTACTAGTGATCTATGACTGATCTGTACATGATCTACA-3′) at room temperature for 1 hour. They were then mixed with Dynabeads M-280 Streptavidin (Life Technologies) in buffer (50 mM Tris-HCl (pH 7.5) containing 100 mM NaCl, 10% glycerol, and 0.5% NP-40) for 1 hour. The beads were washed three times with the same buffer, and the bound proteins were eluted by boiling in SDS sample buffer.

### Antibodies, reagents, cell culture, transfection and immunoblotting

Anti-STING (#3337), anti-phospho-TBK1 (#5483), anti-TBK1 (#3504), anti-phospho-IRF3 (#4947), and anti-IRF3 (#11904) antibodies were purchased from Cell Signaling. Anti-HOIP (SAB2102031), anti-HOIL-1L (NBP1-88301), anti-TRAF2 (558890), and anti-TRAF6 (H-274) antibodies were obtained from Sigma-Aldrich, Novus, BD Pharmingen, and Santa Cruz Biotechnology, respectively. Other antibodies and reagents were obtained as described previously [25]. HEK293T cells were grown in DMEM plus 10% fetal bovine serum, 100 IU ml^−1^ penicillin G and 100 µg ml^−1^ streptomycin. Transfections were performed using Lipofectamine 2000 (Life Technologies). HEK293T cells were co-transfected with pcDNA3.1-STING-FLAG-His_6_ and pXS-Puro, and puromycin-resistant stable clones expressing human STING (293T-STING cells) were selected. SDS-PAGE and western blotting were performed as described previously [25].

### Luciferase reporter assay

The pGL3-IFNβ-promoter-Luc plasmid was kindly provided by Prof. O. Takeuchi (Institute for Virus Research, Kyoto University), and the pGL4-NF-κB-Luc and pGL4-*Renilla*-Luc/TK plasmids were purchased from Promega. Luciferase reporters and pcDNA3.1-myc-cGAS plasmids were co-transfected into 293T-STING cells. At 24 h after transfection, the cells were lysed and the luciferase activity was measured in a GloMax 20/20 luminometer (Promega), using the Dual-Luciferase Reporter Assay System (Promega).

### RNAi

Double-stranded siRNAs for human TRAF2 (SASI_Hs01_00095954), human TRAF6 (SASI_Hs01_00116390), human HOIL-1L (SASI_Hs01_00127700), human HOIP (SASI_Hs01_00167658), and MISSION siRNA Universal Negative Control (SIC-001) were obtained from Sigma-Aldrich. Final 25 nM of siRNAs were transfected to 293T-STING cells by RNAiMAX (Life Technologies) according to the manufacturer’s instructions.

### Quantitative Real-Time PCR

Total RNA was isolated from the indicated cells, using an RNeasy Mini Kit (Qiagen), and subjected to cDNA synthesis, using a SuperScript First-Strand Synthesis System (Life Technologies). Real-time quantitative PCR was performed using a StepOnePlus Real-Time PCR System (Applied Biosystems) and Power SYBR Green PCR Master Mix (Applied Biosystems). The relative mRNA expression of individual genes was calculated using the ΔΔCT method, and normalized to the expression of β-actin. Gene-specific primer sets used in real-time PCR assays were: β-actin, 5′-CCAACCGCGAGAAGATGA-3′ and 5′-CCAGAGGCGTACAGGGATAG-3′; IFNβ, 5′-AGGACAGGATGAACTTTGAC-3′ and 5′-TGATAGACATTAGCCAGGAG-3′; and A20, 5′-CATGCATGCCACTTCTCAGT-3′ and 5′-CATGGGTGTGTCTGTGGAAG-3′.

## Results

### Overall architecture

**Figure 1:**
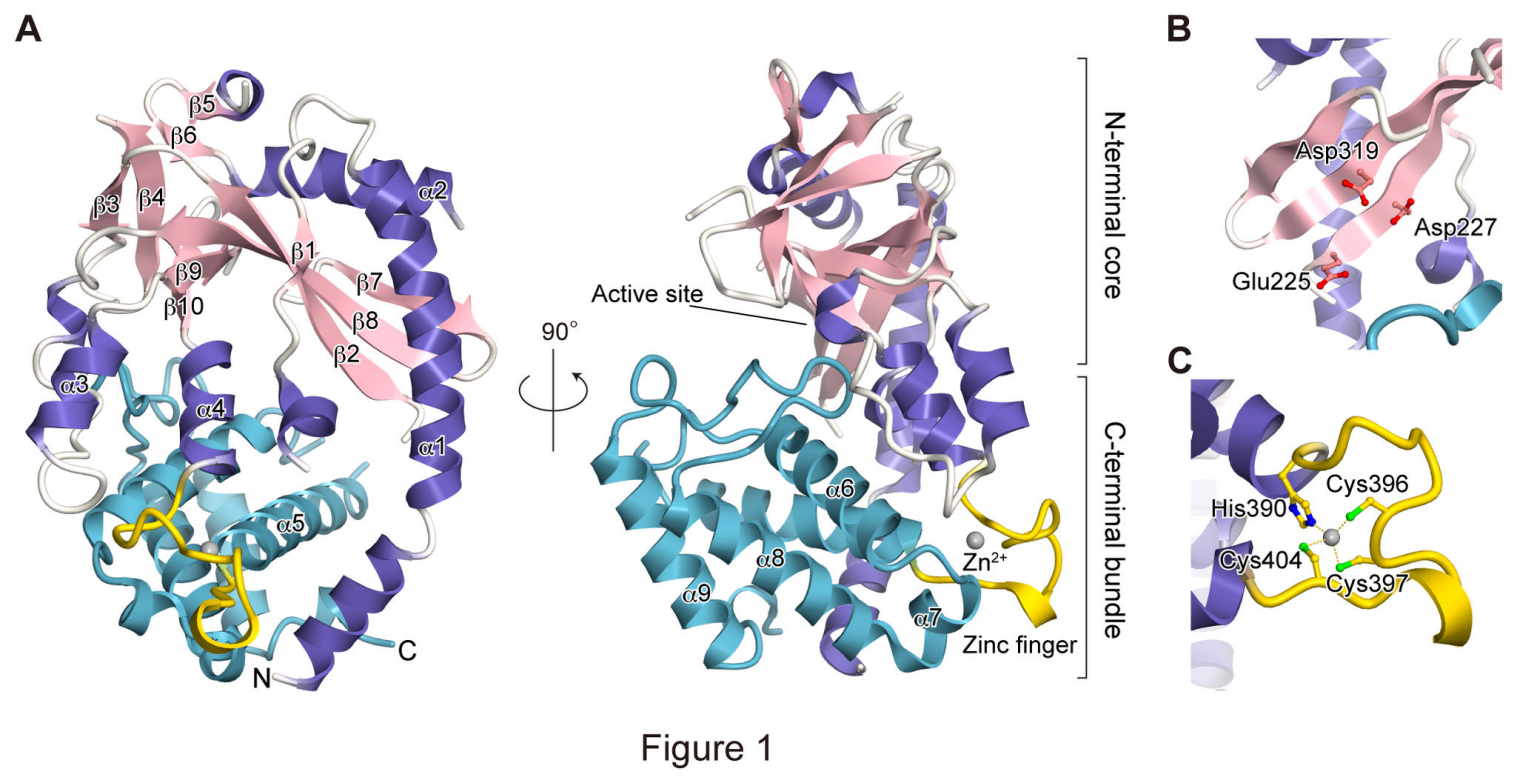
Crystal structure of human cGAS. (A) Overall architecture. The model is shown as a ribbon representation. The α-helices and β-sheets of the N-terminal core are colored violet and pink, respectively. The zinc finger and the C-terminal bundle are colored gold and cyan, respectively. The bound zinc ion is shown as a grey sphere. Secondary structures are numbered. (B) The active site. The catalytic triad residues (Glu225, Asp227 and Asp319) are shown as ball-and-stick models. (C) The zinc finger. The zinc-coordinating residues are shown as ball-and-stick models.

To address the mechanism of immune activation by cGAS, we expressed, purified, and crystallized human cGAS (residues 161–522), including the active site and the double-stranded DNA (dsDNA) binding region [18]. The crystals diffracted to 1.95 Å resolution and belonged to the orthorhombic space group *P*2 _1_2 _1_2, with unit-cell dimensions *a* = 123.5, *b* = 48.31, and *c* = 59.57 Å. Although the space groups are the same between our crystal and that of the previously determined human cGAS, the unit-cell dimensions are different from each other, and the two human cGAS constructs were crystallized in different crystal packing manners [21]. The structure of the human cGAS apo form was determined by molecular replacement, using the structure of the mouse cGAS-dsDNA complex (PDB ID 4K96) as the search model. The final model of human cGAS, except for the disordered regions (residues 218-222, 255-260, 289-305, 363-370 and 426-427), was refined to an R-factor of 20.6% (*R*_free_ = 25.2%). The structure of cGAS adopts the typical nucleotidyl transferase fold, consisting of the N-terminal α/β core and the C-terminal helix bundle (Figure 1A). The N-terminal α/β core contains the centrally-twisted ten-stranded β sheets, surrounded by four helices. The catalytic Glu225, Asp227, and Asp319 residues are located on the centrally-twisted β sheets (Figure 1B). Despite our high resolution structure, electron densities were not observed for the regions between β1 and β2, and between α2 and β7 near the catalytic pocket (Figure 1A), indicating their structural flexibility.

An HCCC type zinc finger (ZnF) exists between the N-terminal core and the C-terminal bundle (Figure 1A). A zinc ion is coordinated by the side chains of His390, Cys396, Cys397 and Cys404, and forms a protruding loop structure (Figure 1C). These zinc-coordinating residues are highly conserved among species, and this ZnF structure is also observed in the mouse and porcine cGAS structures (PDB IDs 4K8V and 4JLX, respectively) [19,20], suggesting its functional importance.

### Structural comparison with the cGAS-dsDNA complex

**Figure 2:**
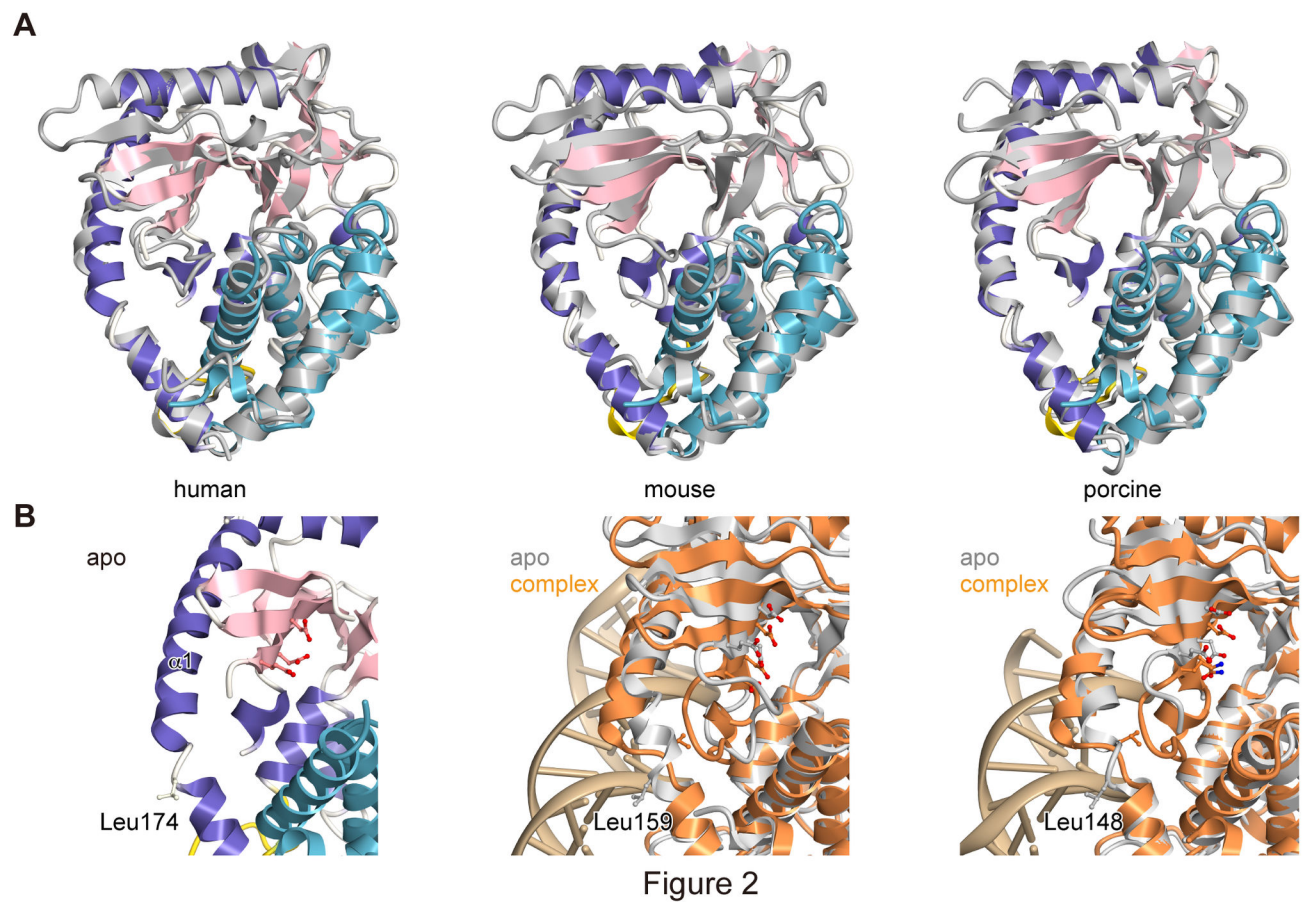
Structural rearrangements of cGAS. (A) Structural comparisons of our human apo-cGAS with the previously determined human (left; PDB 4KM5), mouse (middle; PDB 4K8V) and porcine (right; PDB 4JLX) apo-cGASs. Our human cGAS is shown as a ribbon model, with the same coloring as in Figure 1A. The other cGASs are shown as silver ribbon models. (B) Close-up view of α1 in human apo (left), mouse apo and its complex with 18 bp dsDNA (PDB 4K96) (middle), and porcine apo and its complex with 14 bp dsDNA (PDB 4KB6) (right). The mouse and porcine apo-cGASs are shown as silver ribbon models, and the cGASs complexed with dsDNAs are shown as orange ribbon models. dsDNAs are shown as brown ribbons. The leucine residue on α1 and the catalytic triad are shown as ball-and-stick models.

Recently, the structures of mouse cGAS alone (PDB ID 4K8V) and complexed with an 18 bp dsDNA (PDB ID 4K96), and porcine cGAS alone (PDB ID 4JLX) and complexed with a 14 bp dsDNA (PDB ID 4KB6) were independently determined [19,20]. Our structure of the human cGAS apo form resembles those of the mouse (69% sequence identity, r.m.s. deviation = 1.40 Å for 315 Cα atoms) and porcine (74% sequence identity, r.m.s. deviation = 1.11 Å for 312 Cα atoms) apo proteins (Figure 2A). To further investigate the mechanism of DNA detection by cGAS, we compared the structure of the human cGAS apo form with the mouse and porcine cGAS-dsDNA complex structures. The comparisons revealed that the Leu159 residue in the mouse cGAS and the Leu148 residue in the porcine cGAS (equivalent to Leu174 in human) on α1 are rearranged from the outside to the central β sheet upon dsDNA binding, resulting in the stabilization and activation of the catalytic pocket (Figure 2B) [19,20]. In our structure, Leu174 is oriented toward the solvent, as seen in the mouse and porcine apo structures, and the catalytic pocket is partially disordered. These observations suggested that a similar structural change would occur in human cGAS upon DNA binding for catalytic activation.

**Figure 3:**
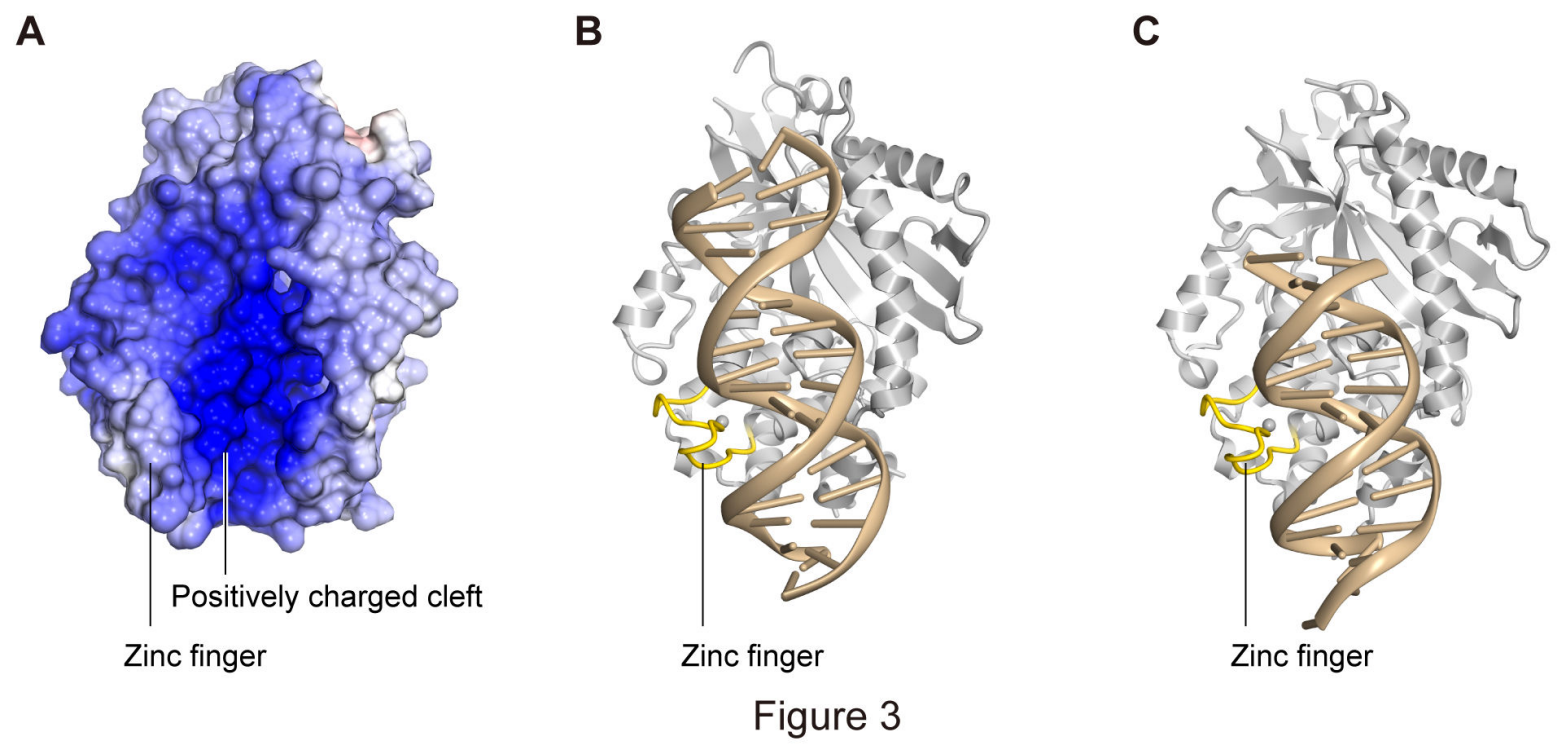
DNA-binding cleft of cGAS. (A) Electrostatic surface potential of human cGAS. (B) (C) Mouse (B) and porcine (C) cGAS complexes with dsDNA. The models are shown as ribbon representations.

**Figure 4:**
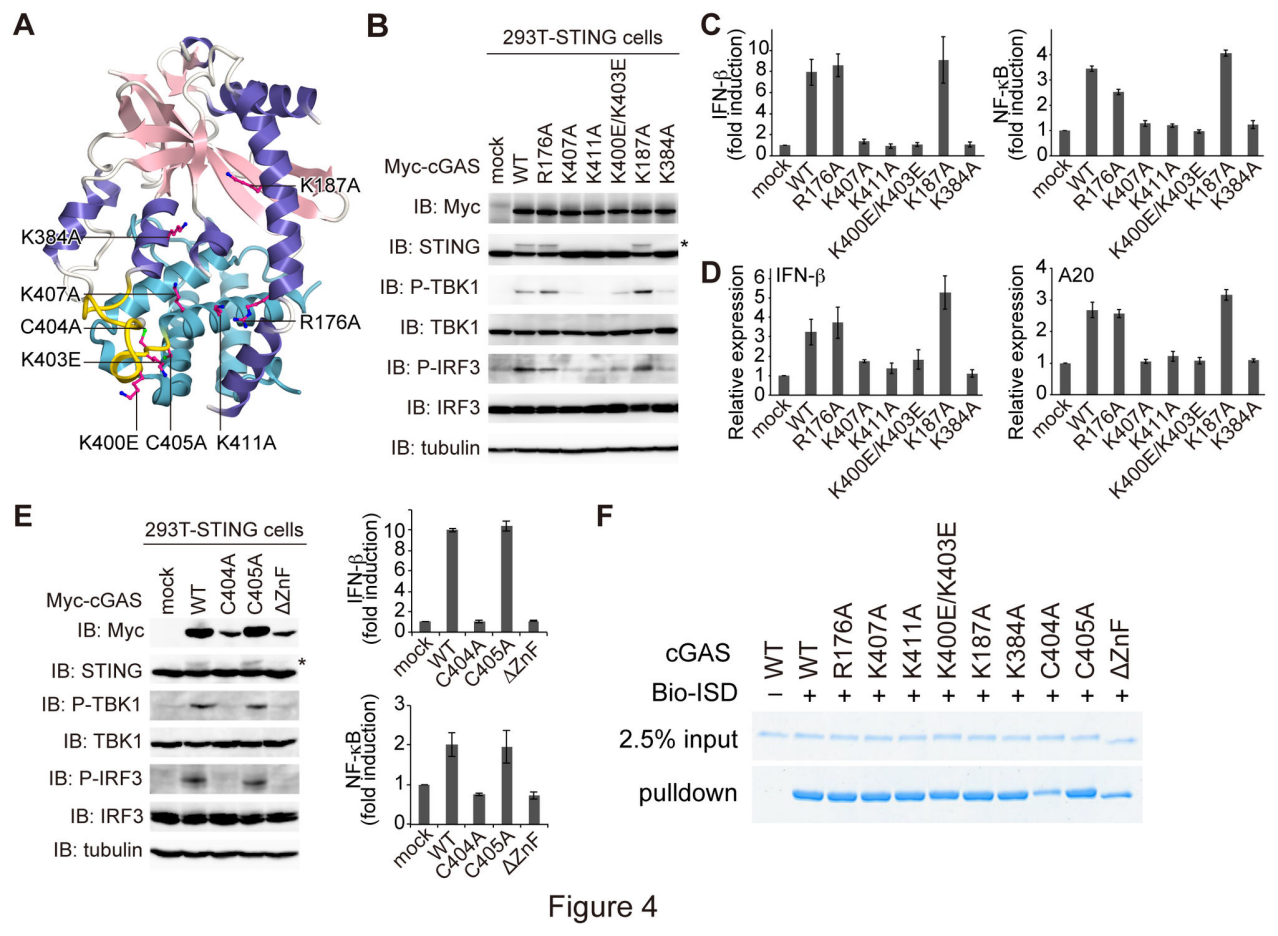
Identification of crucial residues of human cGAS for IRF3 and NF-B activation. (A) Human cGAS mutants used for functional analyses. Human cGAS is shown as a ribbon model, with the same coloring as in Figure 1A. Mutated residues are shown as red ball-and-stick models. (B) cGAS-induced phosphorylation of TBK1, IRF3, and STING. cGAS WT or mutants were expressed in HEK293T cells stably expressing human STING. Cell lysates were analyzed by western blotting, using the indicated antibodies. The asterisk indicates phosphorylated STING. (C) Reporter assays for IFN-β (left panel) and NF-κB (right panel). The cell lysates are the same as in (B), except for the co-expression with reporter plasmids, and were measured for luciferase activities. Luciferase activities are shown as mean ± s.d. (n = 3). (D) Induction of IFN-β and A20 by human cGAS and its mutants. The relative mRNA expression levels of IFN-β (left) and A20 (right) were analyzed by Real-Time PCR using total RNAs isolated from the cells shown in (B). Relative expression levels are shown as mean ± s.d. (n = 3). (E) Immunoblotting for cGAS-induced phosphorylation (left panel), reporter assays for IFN-β (upper panel) and NF-κB (lower panel), the same as (B) and (C), respectively. (F) Pull-down experiment between biotinylated-ISD and human cGAS mutants. Human cGAS mutants were expressed and purified from *E. coli*, and mixed with Streptavidin beads in the presence or absence of biotinylated-ISD. Bound proteins were eluted with SDS sample buffer and analyzed by SDS-PAGE.

As in the mouse and porcine structures, the human cGAS has a concave cleft, mainly formed by α1, α4, α5, and ZnF, on the opposite side of the active site (Figures 1A and 3A). In the structures of the mouse and porcine cGAS complexes with dsDNA, the cleft participates in dsDNA binding (Figure 3B and 3C). Although the dsDNAs used in the mouse and porcine complex structures have different lengths and sequences, the dsDNA recognition mechanism is the same in both structures. This suggested that human cGAS also binds to dsDNA by using the cleft in a similar manner to the mouse and porcine structures, and that the structural features of dsDNA recognition are important for DNA binding by the cGASs. The concave DNA binding cleft of human cGAS is composed of a positively charged cluster of lysine and arginine residues, mainly from α1, α4, and α5 (Figure 3A). These lysine and arginine residues are highly conserved among the cGASs, including the mouse and porcine proteins. In the complex structures of mouse and porcine cGAS with dsDNA, the dsDNA is mainly recognized by the interactions between the side chains of the lysine and arginine residues and the phosphate moieties of the dsDNA. These observations suggested that the positive charges on the cleft are important for DNA binding by human cGAS. Furthermore, the structural comparison revealed a slight difference in the ZnF architecture, which is involved in dsDNA binding [20], between the human cGAS and the mouse and porcine cGASs. In the mouse and porcine structures, the ZnFs are mainly composed of loop structures stabilized by the zinc ion (Figure 3B and 3C) and are similar between the apo and DNA-bound forms [19,20], indicating their rigid structures. In contrast, the ZnF in the human structure forms a partial helix with Lys400 (equivalent to Ser388 and Asp377 in the mouse and porcine proteins, respectively), which is exposed towards the solvent (Figure 4A), suggesting an additional interaction between the ZnF of human cGAS and dsDNA. These observations suggested that human cGAS recognizes dsDNA by using its positively charged cleft and ZnF structure, in a slightly different manner as compared to the other cGASs.

### Functional analyses

In the presence of dsDNA, cGAS produces cGAMP from GTP and ATP, which activates STING as a second messenger [17,18]. Activated STING induces TBK1-dependent phosphorylation of IRF3, resulting in the production of IFN-β [14,15]. To confirm the importance of the positively charged cleft in the STING-dependent immune activation, we mutated the positively charged residues in the DNA-binding cleft or on the ZnF (Figure 4A). We transiently overexpressed the wild type (WT) and mutants of full-length human cGAS in HEK293T cells stably expressing human STING. We then examined the phosphorylation of TBK1 and IRF3 using western blotting analyses, and the induction of IFN-β using luciferase reporter assays and Real-Time PCR. cGAS WT induced the phosphorylation of TBK1, and IRF3 in a STING-dependent manner, as previously reported (Figure 4B) [18]. Immunoblotting for STING revealed that it was also phosphorylated in the presence of cGAS WT (Figure S1). A previous study showed that STING is phosphorylated by activated TBK1 [15], indicating that cGAS induces the phosphorylation of STING following that of TBK1 activation. Despite the similar expression levels of the WT and mutant proteins, the K384A, K407A, K411A, and K400E/K403E mutants abolished the phosphorylation of TBK1 and IRF3 (Figure 4B). These mutants also reduced the induction of IFN-β as compared with cGAS WT (Figure 4C and 4D). Lys384, Lys407, and Lys411 are located in the center of the DNA-binding cleft, while both Lys400 and Lys403 are located on the ZnF (Figure 4A), which would interact with the major groove of dsDNA upon DNA binding, as seen in the complex structures of the mouse and porcine cGASs (Figure 3B and 3C). A previous study showed that the K403E mutant of human cGAS retained the IFN-β production activity [21]. However, our K400E/K403E double-mutant abolished the phosphorylation of TBK1 and IRF3 (Figure 4B). Although Lys400 is not conserved in the mouse and porcine cGASs, these results suggested the importance of Lys400 in the human STING-dependent immune pathway. To confirm the importance of the ZnF motif in the STING-dependent immune activation, we also mutated the cysteine residues on the ZnF or replaced the ZnF (residues 390-405) with a flexible linker, and measured the phosphorylation of TBK1 and IRF3, and the induction of IFN-β. The WT and C405A mutant retained the activities of the STING-dependent signal transduction and the induction of IFN-β while the C404A and ZnF deletion mutants (ΔZnF) abolished these activities (Figure 4E). In the ZnF structure of human cGAS, the zinc ion is coordinated by Cys404, but not Cys405 (Figure 1C and Figure 4A), indicating the importance of the ZnF in the STING-dependent immune activation.

STING was identified as a sensor protein that responds to cytosolic DNA and activates both IRF3 and NF-κB [13,14]. To examine whether cGAS can activate NF-κB in the STING-dependent manner, we also measured NF-κB activation by using luciferase reporter assays and by measuring the level of A20 mRNA, which encodes an NF-κB-inducible deubiquitinase [25]. While cGAS WT was able to activate the NF-κB pathway in the presence of STING, the mutants defective in IFN-β production could not activate the NF-κB signal (Figure 4C and 4D). These observations indicated that human cGAS is involved in the NF-κB activation as well as the IFN-β production through the STING-dependent pathway. Taken together, the positively charged cleft and the ZnF motif of human cGAS are important for STING-dependent signal transduction and immune activation. In addition, we investigated whether the TRAF proteins and LUBAC, essential ubiquitin ligases (E3s) for the canonical NF-κB activation, play an important role in the cGAS-induced NF-κB activation. TRAF proteins and LUBAC, which is composed of HOIL-1L, HOIP and SHARPIN subunits, are E3 ubiquitin ligases that mediate K63- and M1-linked polyubiquitination, respectively [26-28]. We silenced the expression levels of the endogenous TRAF2, TRAF6, HOIL-1L, or HOIP, using respective siRNA and measured the cGAS-induced NF-κB activation. Luciferase reporter assays showed that single knockdown of each ligase had no inhibitory effect on the cGAS-induced NF-κB activation (Figure S2).

To clarify the relationship between DNA binding and cGAS activity, we performed a pull-down assay using biotinylated ISD. Wild-type cGAS and the C405A mutant efficiently bound to biotinylated ISD, while both the C404A and ΔZnF mutants showed remarkably decreased DNA binding affinity (Figure 4F), indicating the importance of ZnF for DNA binding by cGAS. Surprisingly, the K407A, K411A, K400E/K403E, and K384A mutant proteins, which are defective in the immune activation in cells, retained DNA binding ability comparable to that of the cGAS WT *in vitro*. These data suggested that the binding of cGAS to DNA is necessary, but not sufficient, for the conformational change of cGAS and immune activation.

## Discussion

We solved the crystal structure of human cGAS in the apo form. During our ongoing research, the structures of human, mouse and porcine cGAS alone and in complexes with dsDNA were determined, independently [19-21]. Structural comparisons and functional analyses confirmed that the common structural features observed among the species are important for the STING-dependent immune activation by cGAS. Upon DNA binding, the leucine residue (Leu159 and Leu148 in the mouse and porcine proteins, respectively) on helix α1 moves significantly, from the solvent toward the catalytic pocket, to avoid steric hindrance with the phosphate backbone of dsDNA. This movement rearranges the catalytic triad and stabilizes the active site of cGAS [19,20] (Figure 2B). In our apo-structure of human cGAS, Leu174, which corresponds to Leu159 and Leu148 in the mouse and porcine proteins, respectively, is pointed toward the solvent in a similar manner to those in the mouse and porcine apo structures. Leu174 is reportedly crucial for the production of cGAMP and IFN-β in human [20]. These observations indicated that this leucine residue works as a conserved structural switch, which strictly regulates cGAMP production in response to dsDNA binding.

The DNA binding cleft of human cGAS is positively charged, as observed in the mouse and porcine structures [19,20] (Figure 3A). Our mutant analysis revealed that the Lys384, Lys400, Lys407, and Lys411 residues on the positively charged cleft are crucial for the STING-dependent signal transduction and the production of IFN-β (Figure 4B, 4C and 4D). Despite their defective immune activation, these mutants retained the DNA binding ability (Figure 4F), consistent with previous gel-shift analysis results [20]. In addition, the cGAS K407A/K411A double-mutant protein reportedly retained the DNA binding ability, but abolished the production of cGAMP and IFN-β [20]. These mutants defective in the immune activation may bind to DNA in a nonspecific manner using its cluster of the positively charged residues. These results indicated that the interaction between cGAS and dsDNA is not sufficient for the production of cGAMP and the STING-dependent immune activation. Instead, the structural rearrangement of the leucine residue upon dsDNA binding is probably indispensable for these phenomena. cGAS has the highly conserved ZnF motif (Figure 1A and 1C), which is crucial for the STING-dependent immune activation and is partially involved in DNA binding (Figure 4E and 4F) [20,21]. Notably, in the complex structures with dsDNA, the ZnF recognizes the major groove of dsDNA, resulting in the juxtaposition of the dsDNA backbone and the leucine residue on α1 (Figure 3B and 3C). These observations indicated that ZnF reinforces the proper orientation of the dsDNA for the structural rearrangement of the leucine residue. As discussed previously [20], A-form dsRNA has narrow major grooves, while the major grooves of B-form dsDNA are wide enough to accommodate the ZnF motif, thus explaining why cGAS can produce cGAMP in the presence of dsDNA, but not dsRNA. Taken together, the binding between cGAS and dsDNA, but not dsRNA, in the proper orientation is crucial for cGAMP production and immune activation.

Although STING was identified as a sensor protein activating both IRF3 and NF-κB [13], it was unknown whether the cGAS-induced activation of STING is involved in the activation of NF-κB. Our cell-based analyses revealed that cGAS could activate both the NF-κB and IRF3 pathways in a STING-dependent manner (Figure 4C and 4D). Although TRAF proteins and LUBAC are essential for the canonical NF-κB activation [26-28], our knockdown experiments showed that none of these E3 ligases alone were crucial for the cGAS-induced NF-κB activation under our experimental conditions (Figure S2). A recent study showed that these E3 ubiquitin ligases act redundantly to activate a MAVS pathway for the production of IFN-β [29]. These E3 ligases may have redundant functions in the cGAS-induced NF-κB activation as well and further experiments are needed to understand the precise mechanism of the NF-κB activation in the future. Recent reports have revealed that the cGAMP produced by cGAS is linked via an atypical 2′-5′ phosphodiester bond [19,30,31]. This cGAMP binds to STING with higher affinity than cyclic di-GMP, which is a bacterial second messenger, resulting in efficient signal transduction [32]. Taken together, cGAS is a cytosolic DNA receptor that activates both IRF3 and NF-κB by cGAMP production. The STING-dependent immune activation is involved in autoimmune diseases, such as Aicardi-Goutières syndrome and polyarthritis [33,34]. Our structural information for human cGAS will facilitate the development of inhibitors targeting the STING pathway.

## Supporting Information

Figure S1 **Phosphorylation of STING by activated TBK1.** The cell lysates used in Figure 4B were treated with lambda protein phosphatase (λPPase, New England BioLabs), and analyzed by immunoblotting with anti-STING. The upper band of STING was abolished in the presence of λPPase, indicating that the band is phosphorylated STING. *, Phosphorylated STING.
(TIF)

Figure S2 **Human cGAS-induced NF-κB activation is not affected by knockdown of TRAF proteins or LUBAC.** (A) Blotting analyses for E3 ubiquitin ligases, cGAS, and STING. The cell lysates are the same as in Figure 4B, except for the transfection with indicated siRNAs, and were analyzed by western blotting. *, Phosphorylated STING. (B) Reporter assays for IFN-β under the depletion of each E3 ubiquitin ligase. The cell lysates are the same as in (A), and were measured for luciferase activities. Luciferase activities are shown as mean ± s.d. (n = 3).
(TIF)
