# Supplementary figures and images for: Structural and Functional Analyses of DNA-Sensing and Immune Activation by Human cGAS
Source: PLoS One. 2013 Oct 7;8(10):e76983. doi: 10.1371/journal.pone.0076983 (PMC3792152; doi:10.1371/journal.pone.0076983)

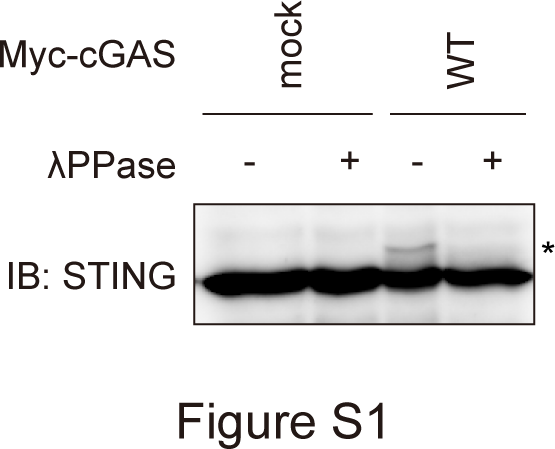

Supplement: Figure S1 — Phosphorylation of STING by activated TBK1. The cell lysates used in Figure 4B were treated with lambda protein phosphatase (λPPase, New England BioLabs), and analyzed by immunoblotting with anti-STING. The upper band of STING was abolished in the presence of λPPase, indicating that the band is phosphorylated STING. *, Phosphorylated STING. (TIF) [file pone.0076983.s001.tif]

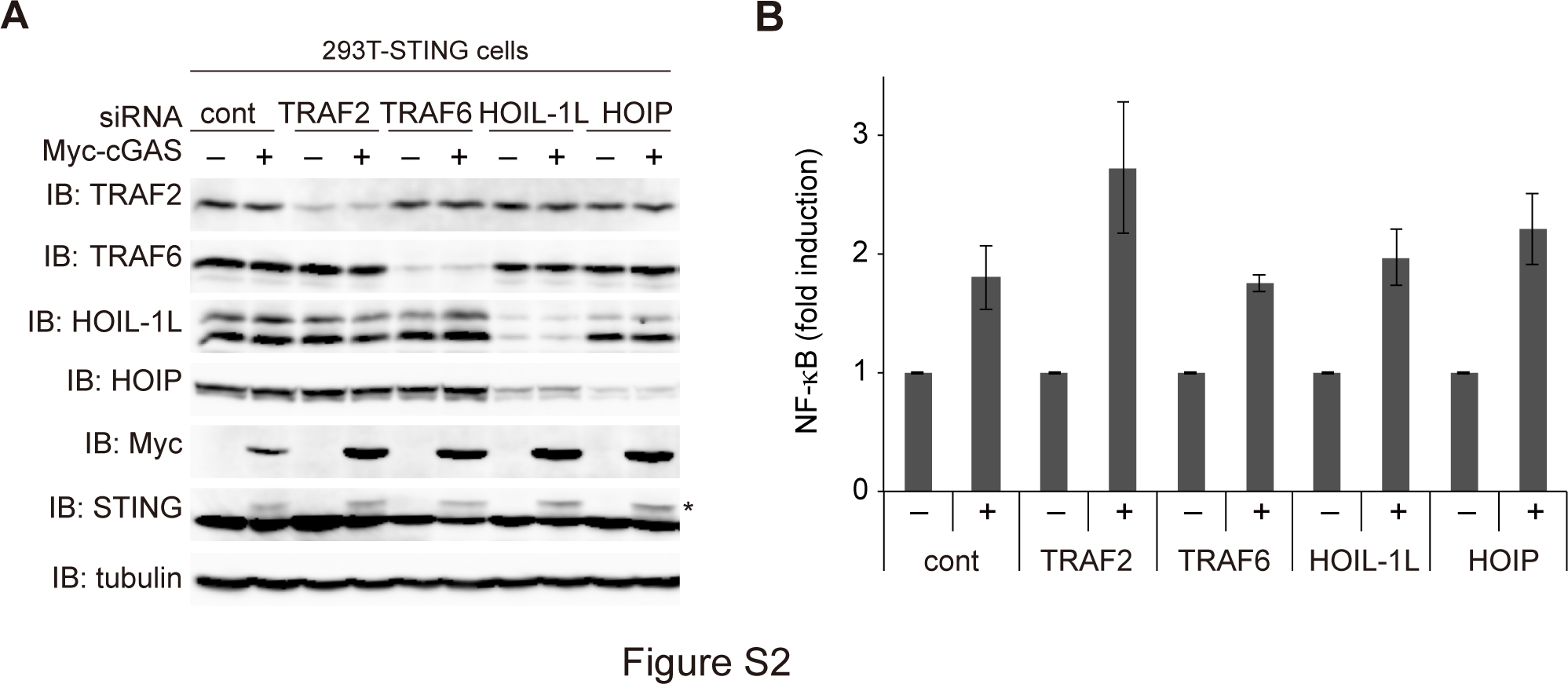

Supplement: Figure S2 — Human cGAS-induced NF-κB activation is not affected by knockdown of TRAF proteins or LUBAC. (A) Blotting analyses for E3 ubiquitin ligases, cGAS, and STING. The cell lysates are the same as in Figure 4B, except for the transfection with indicated siRNAs, and were analyzed by western blotting. *, Phosphorylated STING. (B) Reporter assays for IFN-β under the depletion of each E3 ubiquitin ligase. The cell lysates are the same as in (A), and were measured for luciferase activities. Luciferase activities are shown as mean ± s.d. (n = 3). (TIF) [file pone.0076983.s002.tif]
